# Plastic Deformation Behavior of Bi-Crystal Magnesium Nanopillars with a $\left\{10\bar{1}2\right\}$ Twin Boundary under Compression: Molecular Dynamics Simulations

**DOI:** 10.3390/ma12050750

**Published:** 2019-03-05

**Authors:** Xiaoyue Yang, Shuang Xu, Qingjia Chi

**Affiliations:** Hubei Key Laboratory of Theory and Application of Advanced Materials Mechanics, Wuhan University of Technology, Wuhan 430070, China; yangxiaoyue@whut.edu.cn (X.Y.); qingjia@whut.edu.cn (Q.C.)

**Keywords:** molecular dynamics, twin boundary, magnesium, twining, slip

## Abstract

In this study, molecular dynamics simulations were performed to study the uniaxial compression deformation of bi-crystal magnesium nanopillars with a $\left\{10\bar{1}2\right\}$ twin boundary (TB). The generation and evolution process of internal defects of magnesium nanopillars were analyzed in detail. Simulation results showed that the initial deformation mechanism was mainly caused by the migration of the twin boundary, and the transformation of TB into (basal/prismatic) B/P interface was observed. After that, basal slip as well as pyramidal slip nucleated during the plastic deformation process. Moreover, a competition mechanism between twin boundary migration and basal slip was found. Basal slip can inhibit the migration of the twin boundary, and $\{10\bar{1}1\}\langle 10\bar{1}2\rangle$ twins appear at a certain high strain level (ε = 0.104). In addition, Schmid factor (SF) analysis was conducted to understand the activations of deformation modes.

## 1. Introduction

Recently, magnesium (Mg) alloys have attracted extensive attention due to their exceptional and unique properties, such as low density, high strength, superior damping capacity, and efficient recyclability [1,2,3,4]. However, the deformation behaviors of hexagonal-close-packed (hcp) metal involved numerous twinning activities due to their insufficient slip systems [5,6]. Several types of twins have been reported for Mg alloys, including $\{10\bar{1}1\}\langle 10\bar{1}\bar{2}\rangle$, $\{10\bar{1}2\}\langle 10\bar{1}\bar{1}\rangle$, $\{10\bar{1}3\}\langle 30\bar{3}2\rangle$, $\{10\bar{1}4\}\langle 20\bar{2}1\rangle$, $\{\bar{2}112\}\langle 11\bar{2}3\rangle$, $\{\bar{2}113\}\langle 22\bar{4}3\rangle$, and $\{\bar{2}114\}\langle 22\bar{4}3\rangle$ [7,8]. It is commonly recognized that the $\left\{10\bar{1}2\right\}$ twin is the most common type in hcp metals [9,10,11], which play an important role in the deformation mechanisms of hcp metals [12,13,14].

Twin nucleation and twin growth mechanism has been theoretically studied in hcp metals [15,16,17,18,19,20,21,22]. In addition, Wang et al. [7,18,19] examined the atomic structures of the nucleus of $\left\{10\bar{1}2\right\}$ and $\left\{10\bar{1}1\right\}$ twins in Mg by atomistic simulations. Some studies reported a positive grain size effect, wherein twinning appears to form more easily in larger grains. Experimental observations indicate the mechanism of $\left\{10\bar{1}2\right\}$ twinning growth [20]. Furthermore, we have studied the twinnability of hcp metals at the nanoscale, and presented an analysis on the size effects in deformation twinning [22,23]. Subsequently, we studied the [0001] compression deformation of magnesium nanopillars at various temperatures, and found there was a strong competition mechanism between twins and slip at 300–500 K [24]. Thus, studying the characteristics of twins in Mg may provide a guideline for the design of high-performance Mg, especially the effect of twin boundary (TB) on magnesium.

Moreover, the influence of TB in polycrystalline magnesium has been investigated via experimental methods [25,26,27,28,29], theoretical methods [30,31], and molecular dynamics simulations [32,33,34,35,36]. Experimental studies indicated that internal defect generation and evolution process in nanotwinned Mg can be significantly influenced by TBs [28,29]. The TB itself can act as an obstacle, which can prevent the dislocation from further expanding; it also stores dislocations [37,38,39]. Liu et al. [40] used in situ scanning electron microscopy (SEM) compression test to examine the critical stress associated with basal slip and twinning in bi-crystal Mg with pre-existing $\left\{10\bar{1}2\right\}$ deformation twins. They further interpreted the twinning phenomena and their effects on mechanical behavior. By molecular dynamics simulations, Mei et al. [35] and Song et al. [32] studied the effects of $\left\{10\bar{1}2\right\}$ TB on the compressive and tensile yield strength of Mg nanopillars, respectively.

In addition, it was found that the interaction between dislocation and twin boundary was an important factor to determine the macroscopic properties of nanocrystalline metals [41,42,43]. Many molecular dynamics simulations and discrete dislocation dynamics simulations focused on twin boundary migration and dislocation-twin boundary interactions have been reported [41,42,43,44,45,46]. Mayama et al. [47] investigated the influence of grain boundary in bi-crystal Mg on the activation of slip systems, and showed the calculation of Schmid Factor (SF) for slip systems and twin behavior. Hong et al. [48] studied the role of $\left\{10\bar{1}2\right\}$ twinning characteristics in the deformation behavior of polycrystalline Mg alloy. It was found that the activation of different twin variants is governed by the Schmid law, and consequently, gave rise to a different effect on the deformation.

In order to explore Mg nanopillars with better mechanical properties, the role of $\left\{10\bar{1}2\right\}$ TB on the deformation behaviors of bi-crystal magnesium nanopillars should be studied. In this paper, molecular dynamics simulation was conducted to carry out related research. The deformation mechanisms in bi-crystal Mg were found to be significantly influenced by the activation mode. In addition, the SF was also used to understand the activations of deformation modes. By analyzing the influence of pre-existing TB on the mechanical behavior of Mg nanopillars, it is very important to improve the plasticity of Mg and large-scale application, and it also provides a scientific theoretical basis for the preparation of high-performance nano-polycrystal Mg.

## 2. Methodology

**Figure 1:**
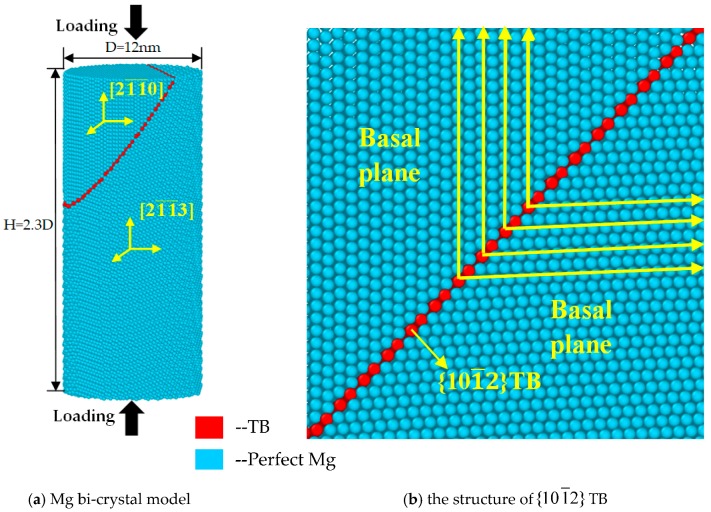
Atomic configurations of Mg bi-crystal nanopillars with $\left\{10\bar{1}2\right\}$ twin boundary (TB). Blue solid circles indicate perfect Mg atoms, while red ones at the $\left\{10\bar{1}2\right\}$ TB.

The most commonly observed type of twin is the $\left\{10\bar{1}2\right\}$ twin; the Mg bi-crystal nanopillar with $\left\{10\bar{1}2\right\}$ TB was built as shown in Figure 1. Compression was performed along [2$\bar{1}\bar{1}$3] and [2$\bar{1}\bar{1}$0] orientation in bi-crystal Mg, and the red atoms represent the $\left\{10\bar{1}2\right\}$ TB. As shown in Figure 1b, the atomic configurations around the $\left\{10\bar{1}2\right\}$ TB were shown. By studying the nanopillars with different cross sections shapes and sizes, we found that they have similar deformation mechanism. Thus, the simulation model with circular cross-sections (with a diameter of 12 nm) was established and the number of atoms was 117,232. The height of nanopillar is about 2.3 times the nanopillar diameter in order to avoid buckling effects. Free surface boundary conditions were applied in x, y, and z directions in simulations. The embedded-atom-method (EAM) potential developed by Liu et al. [49] was used to describe the atomic interactions in Mg. Liu’s potential has previously been used extensively to study the deformation of Mg [18,32,36]. As summarized in [50], this potential agrees well with the density functional theory and experimental measurements, in terms of lattice constants, stacking-fault energy, dislocation nucleation, and twinning behaviors. Furthermore, preliminary simulations using the EAM potential developed by Sun et al. [51] were also conducted, and indicate similar results with Liu’s potential. Thus, only the simulation results based on Liu’s potential were presented in this paper. The simulation was performed in a constant NVT ensemble (i.e., the number of particles N, the volume V, and the temperature T of the model were kept constant) with velocity-Verlet integrator, and a Nosé-Hoover thermostat was used to control the system temperature. The integration time step was fixed at 3 fs.

Simulations were performed by Large-scale Atomic/Molecular Massively Parallel Simulator (LAMMPS) [52], which is a classical molecular dynamics code. At the beginning, initial configurations were relaxed by the conjugate gradient algorithm to obtain a stable simulation configuration. Then, the system was relaxed fully for 120 ps. The initial temperature was set to 10 K, and the NVT ensemble (i.e., the number of particles N, the volume V, and the temperature T of the model were kept constant) was adopted. After that, uniaxial compressions were performed by applying of 0.03% of the compressive strain while keeping fixed the atoms in the top and bottom two layers. In previous studies, the strain rates that have been reported ranged from 5 × 10^7^ /s to 1 × 10^9^ /s [16,17,18,19,20,21,22,30,33,34]. In this work, the strain rate was 1 × 10^9^ /s. This compression and relaxation process was repeated until the maximal strain reached about 25%. To identify the crystal defects in the Mg nanopillars, colors were assigned to the atoms by common neighbor analysis (CNA) method [53] and visualized by the Open Visualization Tool (OVITO 2.9.0) [54]. Atoms on hcp lattice were shown in blue while those at TB were shown in red.

## 3. Simulation Results

**Figure 2:**
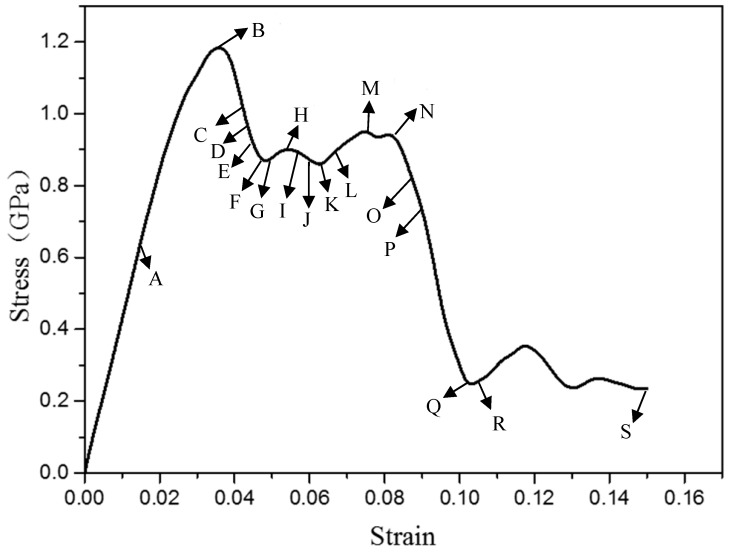
Strain–stress behavior during compression process.

Figure 2 shows the stress-strain curve of an Mg bi-crystal nanopillar under compression. In the elastic deformation process, the stress increases linearly with compressive strain increasing. When the compressive strain reaches 0.035, the stress reaches the highest point (corresponding to point B). Subsequently, the stress dropped suddenly; this indicated that plastic deformation began. In the stage of plastic deformation, the curve was serrated. It was found that the stress curve had an obvious fluctuation stage after point B.

**Figure 3:**
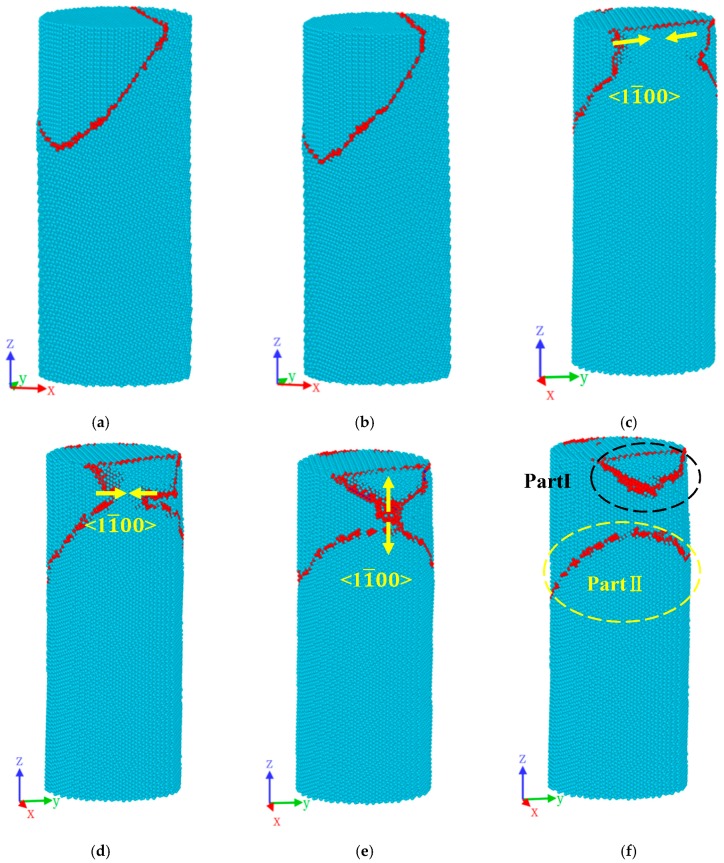
Initial deformation behaviors of bi-crystal Mg nanopillar at different compressive strain level: (**a**) 0.017; (**b**) 0.035; (**c**) 0.038; (**d**) 0.039; (**e**) 0.041; (**f**) 0.044. Snapshots in Figure 3a–f are related to the tagged Points (A–F) in Figure 2.

Figure 3 illustrates the initial microstructure of bi-crystal Mg nanopillar with $\left\{10\bar{1}2\right\}$ TB at different compressive strain conditions when the temperature is 10 K. In the stage of elastic deformation, we found that no obvious migration of the TB occurred during the deformation, as is evident by comparing before (Figure 3a) and after (Figure 3b). After the stress reaches the highest point B (1.19GPa), the TB begins to migrate along the $\langle 1\bar{1}00\rangle$ direction. Compared to Figure 3a,b, the view in Figure 3c–f was rotated 90° around the z-axis in order to show the migration process of the TB. In Figure 3c,d, the TB of the side of the nanopillar gradually migrates and converges (marked by yellow arrow). When the compression strain reaches 0.041, the TB completely converge (Figure 3e) and begin to migrate up and down, respectively, until it is completely disconnected (Figure 3f). Figure 3f shows the atomic configurations of the TB at *ε* = 0.044. Part I of the TB, which is marked in the black dash-lines circle, was constrained on the upper part of the mode, while Part II (in the yellow dash-lines circle) migrates down to the model along the $\langle 1\bar{1}00\rangle$ direction.

**Figure 4:**
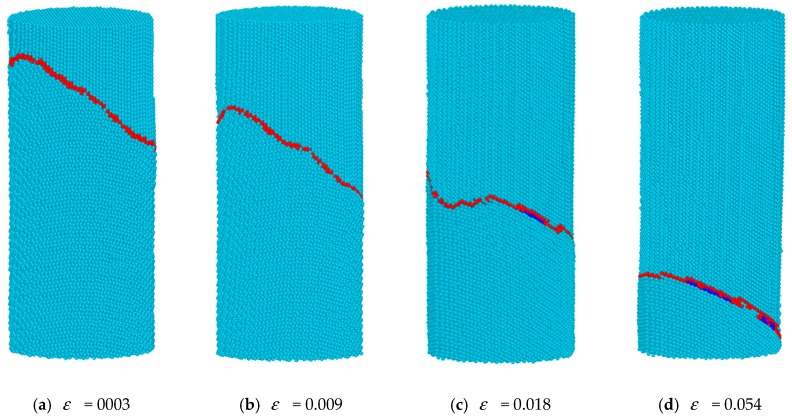
Bi-crystal Mg nanopillar for which atoms in the TB are not fixed.

In order to study whether all the migration mechanism described later was related to what was initial happening in Part I of the TB in Figure 3, we built a model in which the atoms in the TB were not fixed. In Figure 4, it is found that similar mechanism would occur in a geometry in which atoms in the TB were not fixed. This means that the migration mechanism described previously was not affected by the Part I of the TB.

**Figure 5:**
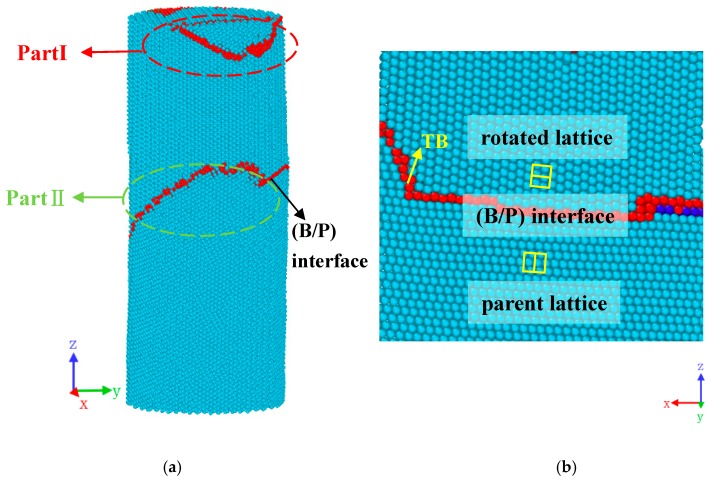
(**a**) Configurations shown for the TB migration at the compressive strain of 0.047. (**b**) A magnified picture of basal/prismatic (B/P) interface.

As the strain increased, the TB structure of Part II migrates down to the middle of the nanopillar in Figure 5a. Moreover, the migration of the TB (Part II) caused lattice reorientation and basal/prismatic (B/P) interface, which connected the rotated crystal with the parent lattice, which can be observed in the nanopillar. Figure 5b displays a slightly magnified picture of the atoms near the B/P interface. In order to make the observation clear, the interface was plotted along the y-axis direction.

**Figure 6:**
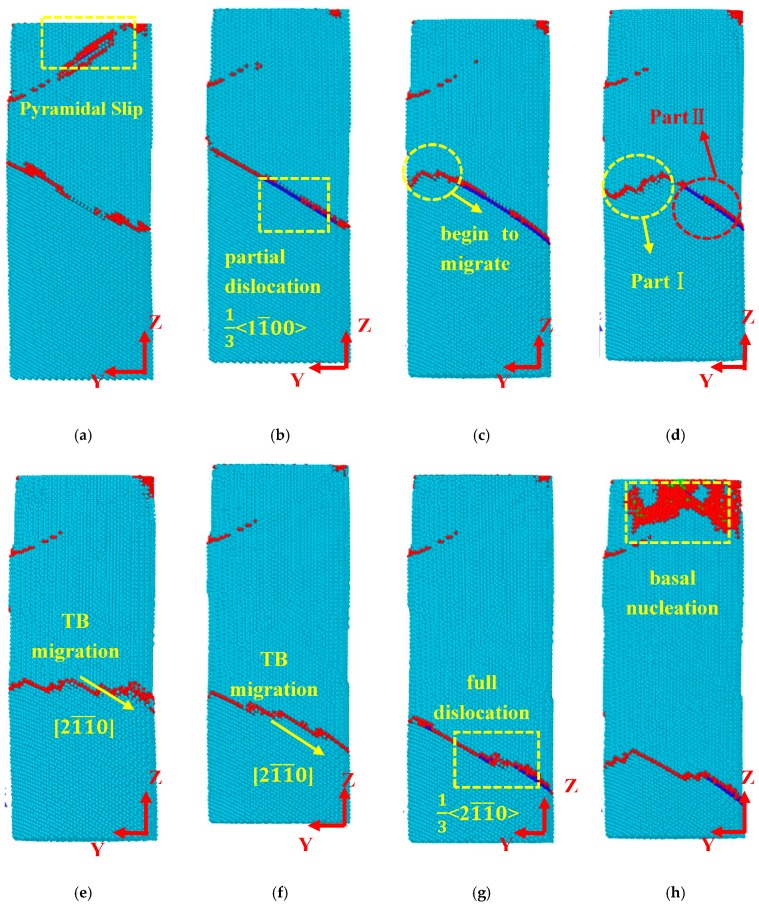
Deformation behaviors during the stress fluctuation stage: (**a**) ε = 0.047; (**b**) ε = 0.053; (**c**) ε = 0.056; (**d**) ε = 0.059; (**e**) ε = 0.062; (**f**) ε = 0.068; (**g**) ε = 0.074; (**h**) ε = 0.083. Images of the atomic configuration in Figure 6a–h are related to the tagged Points (G–N) in Figure 2.

As mention above, the initial plastic deformation was followed by a stress fluctuation stage. Figure 6a shows that the pyramidal slip was observed in the upper part of nanopillar, and the Burgers vector is 1/3 $\langle 1\bar{2}10\rangle$. Figure 6b–d display the microstructure related to Point H, M, and N. As the strain increased to 0.053 (corresponding to Point H), the partial dislocation of basal slip occurred near the TB and the slip direction was $\langle 1\bar{1}00\rangle$ (Figure 6b). Subsequently, TB structure (yellow dash-lines circle), which was not affected by the basal slip began to migrate in Figure 6c. Figure 6c,d show the migration process of TB, and the migration of Part I (yellow dash-lines circle) was found to be prior to Part II (red dash-lines circle). The different migration behaviors were caused by basal slip. The nucleation of basal slip can inhibit the migration of TB to some extent.

At a higher strain level (ε = 0.062), the TB migrated along the $\langle 2\bar{1}\bar{1}0\rangle$ direction quickly (Figure 6e,f). When the strain increased to 0.074 (corresponding to the Point M in Figure 2), full dislocation on the basal plane occurred near the TB (Figure 6g). Thus, the dissociation of basal slip can be showed as follows:

$$\frac{1}{3}[2\bar{1}\bar{1}0]\to \frac{1}{3}[1\bar{1}00]+\frac{1}{3}[10\bar{1}0]$$

As the strain continued to increase, the TB was affected by the basal slip, and the migration speed was very slow. When the strain was 0.083 (corresponding to the Point N in Figure 2), a nucleation of the basal dislocation was observed in the upper part of the nanopillar in Figure 6h.

**Figure 7:**
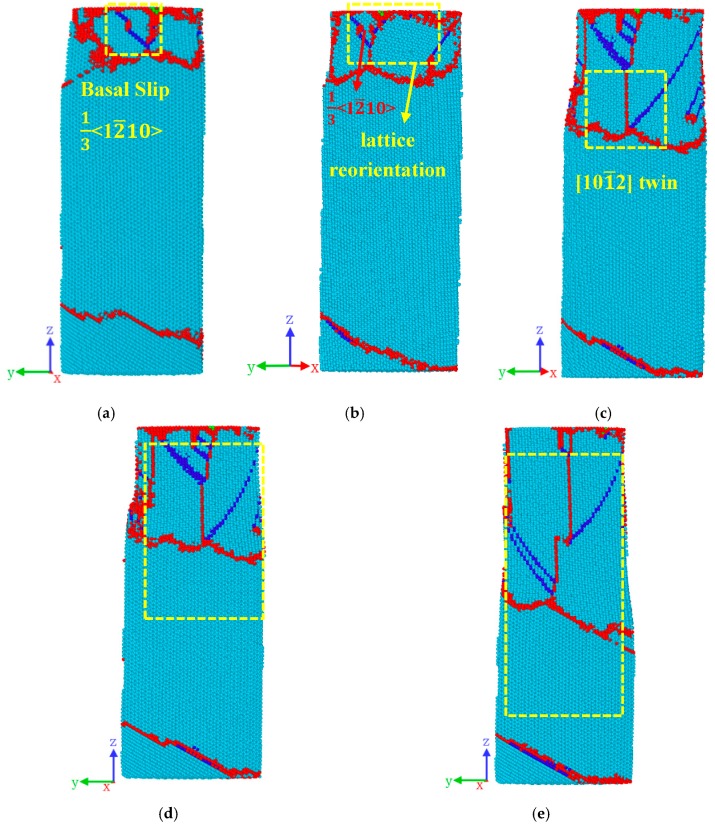
The nucleation and growth process of $\left\{10\bar{1}1\right\}\langle 10\bar{1}2\rangle$ twin at the compressive strains of: (**a**) 0.086; (**b**) 0.089; (**c**) 0.104; (**d**) 0.104; (**e**) 0.149. Snapshots in Figure 7a–e are related to the tagged Points (O–S) in Figure 2.

As can be seen from Figure 7a, the basal dislocations appear in the nanopillar, and the twin boundaries continue to migrate downward. Subsequently, as shown in Figure 7b, the basal slip (Burgers vector is $1/3\langle 1\bar{2}10\rangle$) led to lattice reorientation, and a new TB (highlighted in the yellow frame) was observed in Figure 7a.

**Figure 8:**
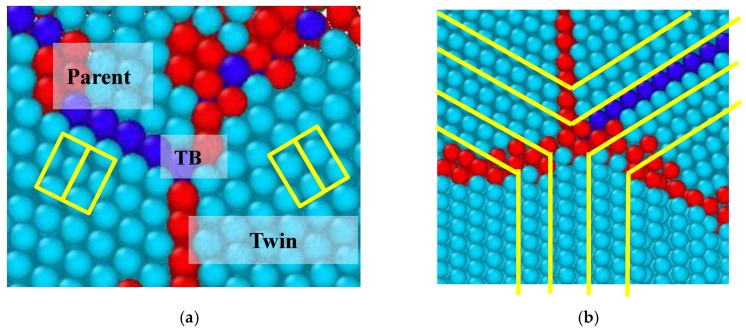
(**a**) TB between the parent lattice and the twined lattice; (**b**) the structure of $\left\{10\bar{1}1\right\}\langle 10\bar{1}2\rangle$ twin.

The new twin grew rapidly (Figure 7c). For a better revealing of the new twin, the region on the twin marked with yellow rectangular box in Figure 7c was selected to perform observation. The corresponding image is presented in Figure 8b. By examining the orientation angle between the matrix and the twinned region, we identified that the new twin was a $\left\{10\bar{1}1\right\}\langle 10\bar{1}2\rangle$ twin. The twin grew gradually while the strain increased, as seen in Figure 7d,e, eventually occupying half of the nanopillar.

## 4. Discussion

In the previous section, simulation results showed that the plastic deformation mechanisms of the bi-crystal Mg nanopillar were mainly TB migration, basal slip, and pyramidal twinning. Compression was performed along [2$\bar{1}\bar{1}$3] and [2$\bar{1}\bar{1}$0] orientation in bi-crystal Mg. Thus, the Schmid factor (SF) of different plastic deformation modes were calculated for [2$\bar{1}\bar{1}$3] and [2$\bar{1}\bar{1}$0], respectively, as shown in Table 1 and Table 2. SF [55] was calculated as follows:

(1) m=cosϕ×cosλ,

where, ϕ is the angle between the loading directions and the slip (or twinning) plane normal and λ is the angle between the loading directions and slip (or twinning) direction. Schmid and Boas [56] established the SF expression:

(2) $$\sigma =\tau_{CRSS}/m,$$

where, σ is the yield stress; $\tau_{CRSS}$ is critical shearing stress (CRSS); m is Schmid factor. It has been widely accepted that the deformation modes are dependent on the SF [57,58]. The deformation mode with a large SF is easier to initiate [59].

Table 1 shows the SF of the plastic deformation modes for [2$\bar{1}\bar{1}$3] compression. The SF values of TB migration and basal slip were 0.43 and 0.38, respectively. TB migration exhibited higher SF values than basal slip. This means that the activation of TB migration may be prior to basal slip, which is in agreement with the simulation results in Section 3. According to Table 2, when the loading was along the axis [2$\bar{1}\bar{1}$0], the SF values of TB migration, basal slip and the nucleation of new twin were 0.34, 0 and 0.37, respectively. Note that TB migration exhibited higher SF than basal slip, which means that the activation of TB migration was prior to basal slip. Besides, the SF value of new twin nucleation had a highest value of 0.37; however, the activation of new twin nucleation was later than TB migration in our simulation. One reason is that compression was performed along [2$\bar{1}\bar{1}$3] (parent) and [2$\bar{1}\bar{1}$0] (twin) orientation for the bi-crystal pillar, and the parent orientation underwent more deformation. That means the analysis of parent orientation can be used to predict the deformation modes of the whole pillar. According to Table 1, the SF of TB migration for [2$\bar{1}\bar{1}$3] compression had the highest SF values of 0.43 and the activation of TB migration was prior to other deformation modes. Therefore, the nucleation of new twin was observed in the later plastic deformation (ε = 0.104). On the other hand, the activation of new twin nucleation may be later than TB migration, which is related to the lack of nucleation source.

Above all, the SF value can be used to predict the activation of TB migration and basal slip. In this work, the TB migrates in the initial plastic process, and the occurrence of basal slip can inhibit the migration of the TB. However, Liu et al. [40] conducted in situ SEM micropillar compression, and found that multiple basal slip events were observed in the middle of the pillar; no obvious migration of the TB occurs during the deformation. The difference between experiment and simulation was because the external environment in experiment and the impurities inside the specimen may contribute to the nucleation of basal slip, and the basal slip inhibits the migration of the TB at the beginning. Simulation results in this work have shown that the nucleation of the basal dislocation has a significant impact on the migration of the TB.

## 5. Conclusions

In this work, molecular dynamics simulations were carried out to study the plastic deformation behaviors of bi-crystal Mg nanopillars with a $\left\{10\bar{1}2\right\}$ twin boundary. Schmid factor (SF) of the deformation modes with two different uniaxial compression loading axis was also calculated. The main results can be summarized as follows.

1. The initial plastic deformation was dominated by the migration of the twin boundary (TB), and there is a mechanism for basal/prismatic transformation.
2. Obvious stress fluctuation during the plastic deformation was caused by competition between TB migration and basal slip. Moreover, pyramidal slip was observed.
3. At a higher strain level (ε = 0.104), the $\left\{10\bar{1}1\right\}\langle 10\bar{1}2\rangle$, twin formation was observed due to the lattice reorientation, and it grew rapidly.

Furthermore, simulations results show that other factors, such as temperature and boundary conditions, may affect the plastic deformation mechanism of the bi-crystal Mg nanopillars. We will conduct simulations and discussions on these influence factors in further work.

## Figures and Tables

**Table 1:** Schmid factor (SF) of plastic deformation modes with loading axis [2$\bar{1}\bar{1}$3].

Deformation Modes	Slip Plane	Slip Direction	SF
TB migration	$\left\{10\bar{1}2\right\}$	$\langle 1\bar{1}00\rangle$	0.43
basal slip	{0001}	$\langle 2\bar{1}\bar{1}0\rangle$	0.38

**Table 2:** Schmid factor (SF) of plastic deformation modes with loading axis [2$\bar{1}\bar{1}$0].

Deformation Modes	Slip Plane	Slip Direction	SF
TB migration	$\left\{10\bar{1}2\right\}$	$\langle 1\bar{1}00\rangle$	0.34
basal slip	{0001}	$\langle 2\bar{1}\bar{1}0\rangle$	0
new twin	$\left\{10\bar{1}1\right\}$	$\langle 10\bar{1}2\rangle$	0.37
